# Supplementary material for: Swine influenza A virus infection dynamics and evolution in intensive pig production systems
Source: Virus Evol. 2024 Feb 27;10(1):veae017. doi: 10.1093/ve/veae017 (PMC10930190; doi:10.1093/ve/veae017)
Supplement: veae017_Supp [file veae017_supp.zip › suppl_data/Supporting Information.docx]

**Supporting Information**

**Supplementary Table S1. Primers utilised for amplicon-based Illumina sequencing.**

Plain font - adapter sequences, Bold font – conserved termini

**
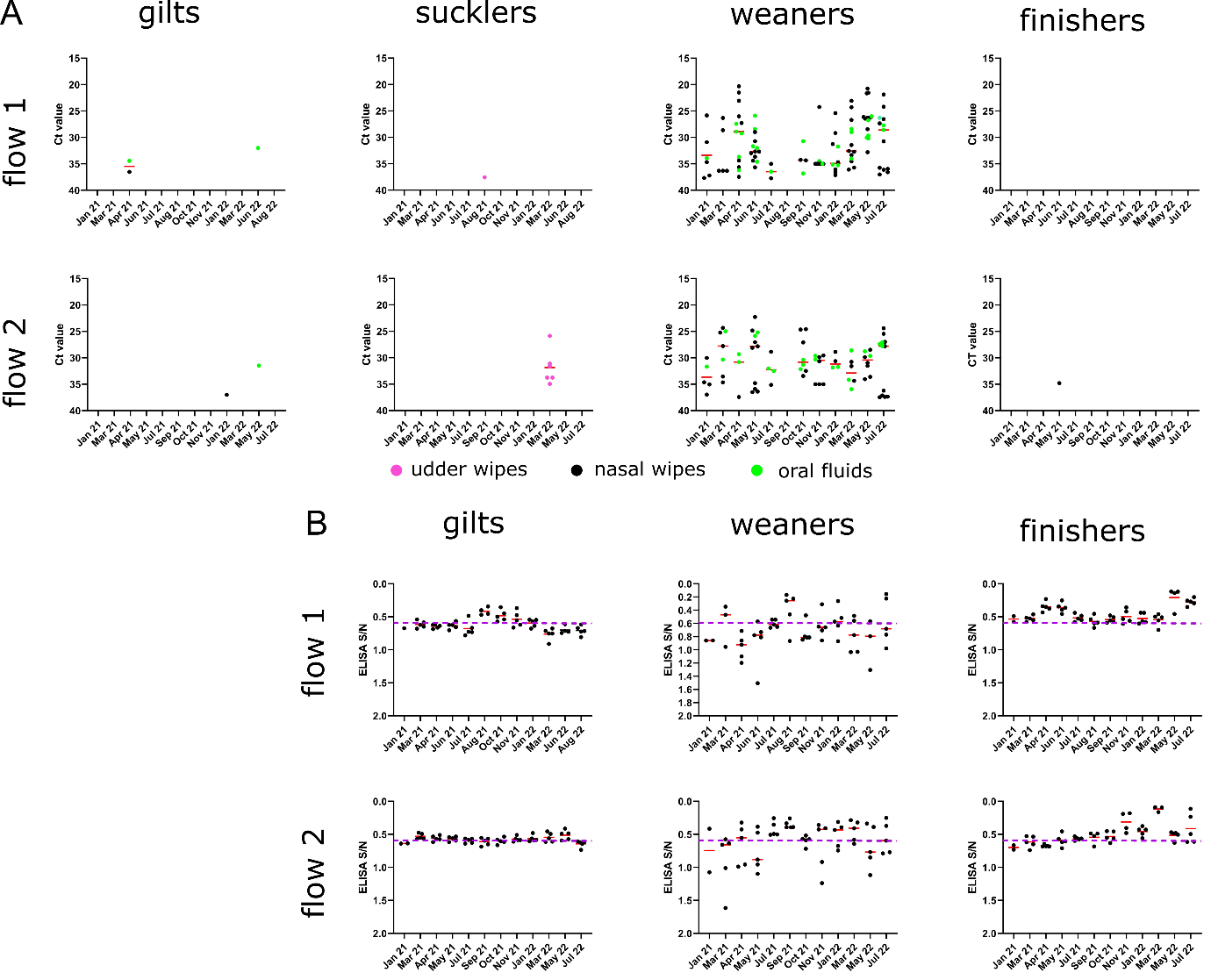
**

**Supplementary Fig S1. Comparison of viral prevalence with antibody status.** (A) Nasal wipes, udder wipes and oral fluids were tested for the presence of swIAV by RRT-PCR and plotted as individual ct values. Data points coloured according to sample type: udder wipes (pink), nasal wipes (black), oral fluids (green), with mean values indicated by red bars. (B) Oral fluid samples were tested for the presence of influenza NP antibodies by a blocking ELISA and plotted as individual S/N ratios (black circles) with mean values indicated by red bars. S/N ratios ≤0.6 are considered antibody positive. The dashed purple line indicates the 0.6 S/N ratio cut-off value.

**Supplementary Table S2. Comparison of swIAV HA and NA amino acid sequences from clinical material and egg passage 1 (E1) isolates.**
